# The effect of abdominal bracing on respiration during a lifting task: a cross-sectional study

**DOI:** 10.1186/s13102-023-00729-w

**Published:** 2023-09-15

**Authors:** Martin Sembera, Andrew Busch, Alena Kobesova, Barbora Hanychova, Jan Sulc, Pavel Kolar

**Affiliations:** 1 Department of Rehabilitation and Sports Medicine, Second Medical Faculty, Charles University and University Hospital Motol, Prague, Czech Republic; 2 Department of Health and Human Kinetics, Ohio Wesleyan University, Delaware, OH USA

**Keywords:** M-mode ultrasonography, Diaphragm, Breathing, Abdominal bracing, Spinal stabilization

## Abstract

**Background:**

Abdominal bracing is a maneuver widely used by rehabilitation specialists and sports trainers to improve spinal stability. This study aimed to investigate how lifting tasks with and without abdominal bracing affect the respiratory function of the diaphragm.

**Methods:**

M-mode ultrasonographic assessment of diaphragmatic motion combined with spirometry was performed on 31 healthy adults. Participants were asked to breathe continuously whilst lifting a load with spontaneous abdominal muscle contraction (natural loaded breathing) and abdominal bracing (AB loaded breathing).

**Results:**

Pearson’s correlations revealed strong correlations between ultrasonography and spirometry measures (*p* < 0.001) for all types of breathing: tidal breathing (*r* = 0.709, *r*^*2*^ = 0.503), natural loaded breathing (*r* = 0.731, *r*^*2*^ = 0.534) and AB loaded breathing (*r* = 0.795, *r*^*2*^ = 0.632). Using paired-samples *t*-tests, the natural loaded breathing ultrasonography revealed more caudal diaphragm positions during inspiration (*p* < 0.001) but not during expiration (*p* = .101). Spirometry demonstrated lower lung volumes (L) at the end of inspiration and expiration (*p* < 0.001), with no changes in total lung volume (*p* = 0.06). The AB loaded breathing ultrasonography revealed more caudal diaphragm positions during inspiration (*p* = 0.002) but not during expiration (*p* = 0.05). Spirometry demonstrated lower lung volumes at the end of inspiration (*p* < 0.001), expiration (*p* = 0.002), and total lung volumes (*p* = 0.019).

**Conclusion:**

This study demonstrated that abdominal bracing performed during a lifting task reduces lung volume despite an increase in diaphragmatic motion. Diaphragm excursions strongly correlate with lung volumes even under postural loading.

**Trial registration:**

The study was prospectively registered on 8 April 2021 at ClinicalTrials.gov with identification number NCT04841109.

## Background

The diaphragm is the first muscle activated during inspiration [1], and its function is closely related to changes in lung volumes. The diaphragm is responsible for 60–80% of the inspiratory work [2], and its contribution to lung volume changes is estimated between 75–90% [3–5]. At higher lung volumes, the diaphragm's ability to generate force decreases as its muscle fibers shorten and change their orientation [6–8], resulting in its lower contribution to changes in tidal volumes and greater involvement of the extra-diaphragmatic inspiratory muscles (EIMs) [3–5]. In addition to the respiratory function, the diaphragm is involved in the postural stabilization of the spine by increasing intra-abdominal pressure (IAP) [6–8]. The diaphragm can perform these two actions simultaneously. When breathing in a posturally challenging situation, its EMG activity is higher compared to tidal breathing [6, 7], and the diaphragm achieves a more caudal position with greater respiratory excursions [9].

Along with the diaphragm, the muscles of the abdominal wall are mostly responsible for regulating IAP [6, 10], which is important not only for spinal stability but also for maintaining sufficient ventilation. If the tone of the abdominal muscles is impaired, the diaphragm descends, and tidal volume decreases substantially [11, 12]. A natural increase in the activity of all abdominal wall muscles is associated with lifting and lowering a load [13]. Voluntary isometric contraction of the abdominal muscles before and during any loaded exercise is also known as abdominal bracing (AB) [14, 15]. Several studies [14–17] have consistently demonstrated that AB increases spinal stability, which is also an effective technique for stabilizing the spine when lifting weights [18]. As it is believed that spinal instability may be one of the causes of low back pain [19], stabilization maneuvers are commonly used in rehabilitation and training programs [20, 21]. In comparison to other maneuvers such as abdominal hollowing, AB has demonstrated a superior improvement in spinal stability [16, 22].

Lifting weights, as part of resistance training, is widely recommended in pulmonary rehabilitation for patients with respiratory disorders [23, 24], including those with post-COVID-19 syndrome [25]. One of the main symptoms of these conditions is dyspnea [26–28], which is associated with increased respiratory effort, decreased ventilation, or both [29]. Therefore, it seems important to investigate how lung volumes change during postural loading with abdominal bracing in order to determine whether stabilization maneuvers may increase the risk of dyspnea in these patients.

During tidal breathing, the relation between diaphragmatic excursions and inspired volume has been shown to be linear [30]. With increased ventilation, the respiratory activity of the diaphragm may conflict with its postural function, resulting in the suppression of the latter [31]. However, it has not yet been reported whether sufficient ventilation can be maintained at higher postural demands. Therefore this study aimed to investigate how diaphragm movement correlates with lung volumes when lifting a load (natural loaded breathing) and when lifting a load with abdominal muscle preactivation through the AB maneuver (AB loaded breathing). We hypothesized that lung volumes would not correlate during both natural loaded breathing and AB loaded breathing with diaphragmatic excursions; as the postural demands on the diaphragm increase, the EIMs should compensate for its respiratory function.

## Methods

### Participants

In this study, 31 healthy adults (average age = 28.7 ± 5.8 years) from the general population, including both athletic and non-athletic individuals, were recruited via social media. The procedures were explained in detail, and signed informed consent was obtained from each participant. Table 1 displays the demographic characteristics of the study group. Similarly to other studies evaluating the function of the diaphragm [32–34], the following excluding criteria were applied: low back pain, previous abdominal or spine surgery, respiratory or musculoskeletal disorder (e.g., scoliosis, chest deformities, ankylosing spondylitis), any symptoms of any kind of disease, medical/surgical procedure or trauma within four weeks before initiation of the study, pregnancy and waist to height ratio (WHtR) greater than 0.59. All procedures were performed in accordance with the 1964 Declaration of Helsinki and were approved by the institutional ethical committee (Ref. No. EK-237/21).

**Table 1:** Descriptive statistics of participants (Mean ± Standard Deviation)

Participants	All (*n* = 31)	Males (*n* = 11)	Females (*n* = 20)
Age (y)	28.7 ± 5.8	28.4 ± 5.0	28.9 ± 6.4
Height (cm)	173.2 ± 8.5	181.0 ± 6.0	169.0. ± 6.4
Weight (kg)	66.2 ± 9.2	74.1 ± 8.0	61.9 ± 6.8
Waist Circumference (cm)	74.1 ± 5.9	78.9 ± 6.2	71.5 ± 3.9
Waist to Height Ratio	0.43 ± 0.03	0.44 ± 0.03	0.42 ± 0.02
Body Mass Index	22.0 ± 1.6	22.6 ± 1.7	21.6 ± 1.4

### Instrumentation

The methods used in this study are identical to those already published in our previous article [35].

#### M-mode ultrasonography

The ultrasonographic examination was performed with Toshiba (Canon Medical Systems Corporation, Otawara, Japan) Aplio i600 ultrasound system by a single experienced ultrasound operator. The movement of the diaphragm was assessed in the M-mode, which is used to track the motion of a given point in time. M-mode assessment of the diaphragm has been described in several studies [36–39]. A low frequency convex 3.5 MHz transducer was placed in the subcostal region between the midclavicular and anterior axillary lines with the probe tilted cranially, medially, and dorsally to scan the posterior third of the right hemidiaphragm perpendicularly. The right subcostal approach provides a good visualization to assess the movement of the hemidiaphragm, as the liver serves as an acoustic window. In contrast, imaging of the left hemidiaphragm is more difficult because the air in the gastrointestinal tract can interfere with visualization, and therefore a smaller acoustic window through the spleen must be used. For deeper breathing, the left hemidiaphragm is not possible to visualize at all in a large proportion of individuals [38].

**Fig. 1:**
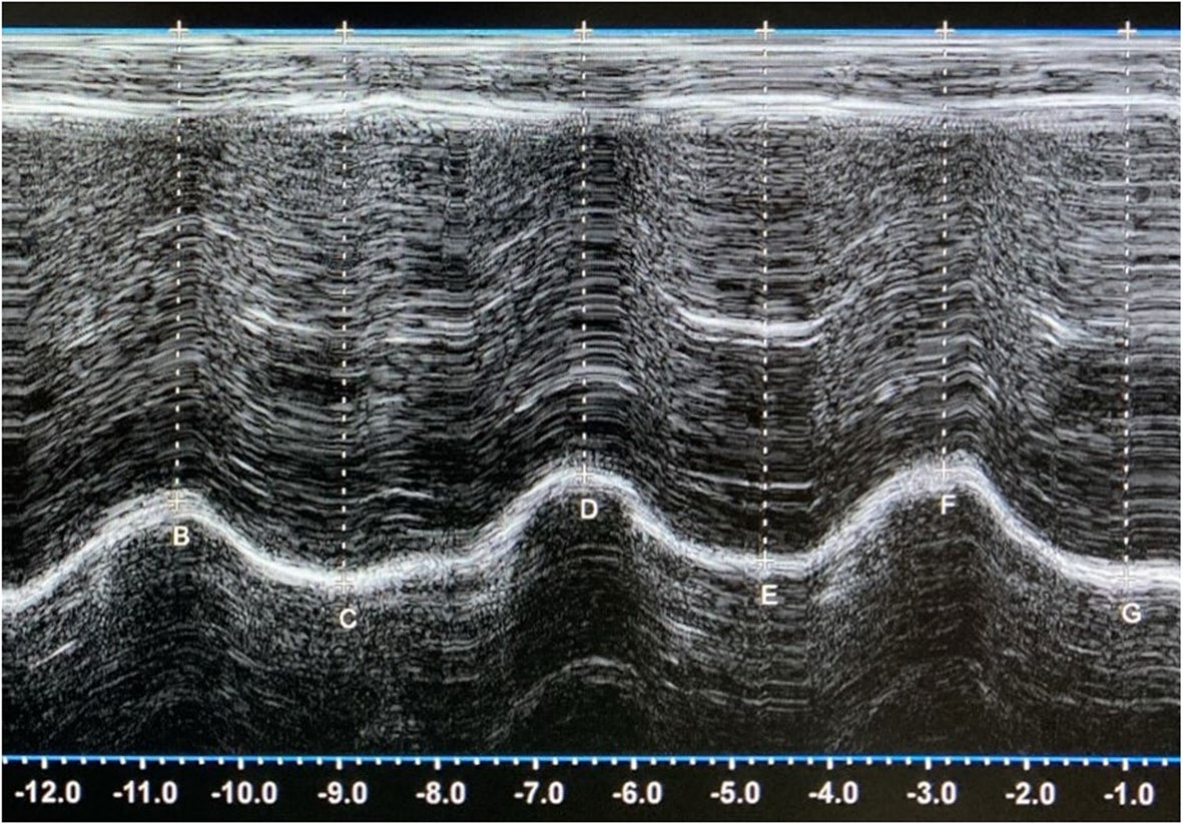
M-mode ultrasonographic image of the diaphragm motion. The cursor is placed on the peak of the curve at the end-inspiratory (upper peaks B, D, F) and end-expiratory (lower peaks C, E, G) phase of breathing with a vertical line to the baseline

In the M-mode trace, the diaphragm is shown as an echogenic line. The position of the diaphragm was determined as the distance between the inspiratory peak and the expiratory peak of the curve from the probe (Fig. 1). This distance was measured vertically from the center of the echogenic line to the baseline. Excursions were then calculated as the difference between the end-expiratory and end-inspiratory positions. All ultrasonographic measurements were reported in millimeters.

#### Spirometry

**Fig. 2:**
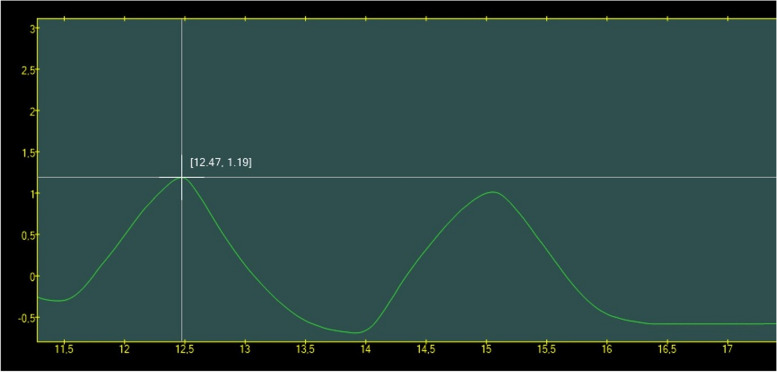
Spirometric time-volume curve displayed in Grapher software. The vertical axis represents the lung volume (in liters), and the horizontal axis represents the time (in seconds). Placing the cursor on the spirometric curve allows us to determine the exact value of the volume at a given time

Lung volumes were measured using a portable spirometer Jaeger MasterScope (VIASYS Healthcare, Hoechberg, Germany) with an original heated pneumotachograph. The initial calibration was performed with a one-liter pump in MasterScope software, followed by a second calibration using the original software called BreathRecorder, described in previous studies [9, 40, 41]. Raw flow-time data were stored directly on the spirometer's hard disk, while the flow signal was integrated to obtain a time-volume signal. All records were corrected for body temperature and ambient pressure saturated with water vapor (BTPS) to increase measurement accuracy. To analyze the recorded spirometry data was used original Grapher software [9, 41]. In this software, we can see any changes in time using a special cursor (Fig. 2). Therefore, any specific time-volume records of inspiratory/expiratory volume changes were accurately measured.

### Procedures

Participants were asked not to eat for at least 1.5 h before the procedure. All examinations were performed in the same quiet room with a stable temperature by the same operators, who were blinded to the results of other assessments. Ultrasonographic and spirometric examinations were performed simultaneously with the subjects in a standing position. Subjects stood with their feet shoulder-width apart, elbows close to their body, and both hands placed on the handle of one kettlebell. They were instructed to lift the kettlebell only by bending their elbows to avoid tilting the trunk and loss of ultrasound imaging of the diaphragm. The weight of the kettlebell was chosen to be close to 20% of the subject's body weight. The lowest kettlebell weight used in this study was 10 kg, and the highest weight was 18 kg. The ultrasound probe was positioned in the right subcostal region and directed to obtain the best possible view of the right hemidiaphragm. The subject was then given a nose-clip, and a pneumotachograph mouthpiece was put in his or her mouth.

The procedure began with a deep inspiration followed by a sharp expiration, which was used as a time marker for both records. One recording lasted up to 20 s. Every procedure was repeated three times in all patients, and the average values of these measurements were then calculated. Diaphragm motion and lung volumes were measured in the following two scenarios:

1. Natural loaded breathing scenario (N-LBS): two tidal inspirations and expirations, then the kettlebell was lifted, followed by two inspirations and expirations while the kettlebell was held.
2. AB loaded breathing scenario (AB-LBS): two tidal inspirations and expirations, then the participants were instructed to contract the abdominal muscles and lift the kettlebell, followed by two inspirations and expirations while holding the kettlebell and having the abdominal wall tensed.

### Statistical analysis

Descriptive statistics were calculated for all variables. Data are mean ± standard deviation (SD) unless otherwise stated. Univariate outliers were assessed for each dependent variable by calculating z-scores using complete data for all scenarios (n = 31). Normality was assessed using ± 1.96 as the cutoff for the absolute z-score skew and kurtosis (respectively) for each variable [42]. Results evidenced three probable-outliers according to z-score values greater than 2.58, which occurred within variables that were not normally distributed. These outliers were handled by winsorization; where the outlier retained its rank value and was replaced with the next largest value [43]. This process improved the normality of those variables to within the acceptable range, where no absolute z-scores were larger than ± 1.96 for skew or kurtosis after the correction of the outliers.

The reliability of the ultrasonography and spirometry measures was calculated from averaging measurements of two tidal breaths recorded at different time points for each subject. Table 2 presents intraclass correlation coefficient estimates (ICC_2,k_), 95% confidence intervals, and standard error of measurement (SEM) calculated from the tidal inspiration and expiration values. The ICC’s were calculated based on a mean-rating (k = 3), absolute-agreement, 2-way random-effects model. Reliability was interpreted as poor (< 0.5), moderate (0.5 – 0.75), good (0.75 – 0.9), and excellent (> 0.9) [44]. Pearson’s correlations were used to assess the relationship between diaphragm excursions and lung volumes for each scenario described, and paired-samples t-tests were used to determine changes in the diaphragm position and lung volumes for each scenario. Power analysis, using G*Power 3.1, indicated an 80% chance of detecting a strong correlation of 0.50 in 29 subjects (two-tailed), and 27 subjects were needed to achieve a medium effect size of 0.5 in paired t-tests (one-tailed), with statistical significance determined a priori at *p* < 0.05. When relevant, Bonferroni corrections were utilized when testing multiple hypotheses. The strength of the correlations was interpreted as weak (< 0.30), moderate (0.30–0.50), or strong (> 0.50), and effect sizes were interpreted as small (< 0.2), medium (0.5), or large (> 0.8) [45]. All data analyses were conducted using the Statistical Package for the Social Sciences (SPSS version 28.0 for Mac; IMB Corp, Armonk, NY).

**Table 2:** Intraclass Correlation Coefficients of ultrasonography and spirometric values during tidal inspiration and expiration (ICC _2, k_)

			**95% Confidence Interval**			**F Test With True Value 0**		
**Measure**		**ICC**	**Lower Bound**	**Upper Bound**	**SEM**	**Value**	***df***	**Sig**
Ultrasonography	Inspiration	.985^b^	.970	.993	1.68	71.63	30	< .001
	Expiration	.989^b^	.978	.995	1.54	93.40	30	< .001
Spirometry	Inspiration	.918^b^	.829	.960	0.23	11.86	30	< .001
	Expiration	.759^a^	.500	.884	0.29	4.09	30	< .001

Note: *ICC* Intraclass Correlation Coefficient

*SEM* Standard Error of Measurement

^a^Denotes: Good reliability

^b^Denotes: Excellent reliability

## Results

### Hypothesis testing

Pearson’s correlations demonstrated strong statistically significant positive relationships between the ultrasonography and spirometry measures for all types of breathing: tidal breathing: *r*(29) = 0.709, *p* < 0.001; natural loaded breathing: *r*(29) = 0.731, *p* < 0.001; and AB loaded breathing: *r*(29) = 0.795, *p* < 0.001 (see Table 3).

**Table 3:** Correlations between Ultrasonographic diaphragm excursions and Spirometry lung volumes during different types of breathing. Values are Mean [Standard Deviation]

Type of Breathing	Ultrasonography (mm)	Spirometry (L)	Pearson r	r^2^	Sig
Tidal Breathing	18.74 (5.74)	1.74 (0.73)	.709	.503	< .001*
Natural Loaded Breathing	21.46 (7.26)	1.65 (0.63)	.731	.534	< .001*
AB Loaded Breathing	20.41 (9.52)	1.55 (0.68)	.795	.632	< .001*

Note: *mm* Millimeter, *L* Liter

*AB *Abdominal bracing

^*^Statistically significant (2-tailed) correlation (*P* < 0.01)

**Fig. 3:**
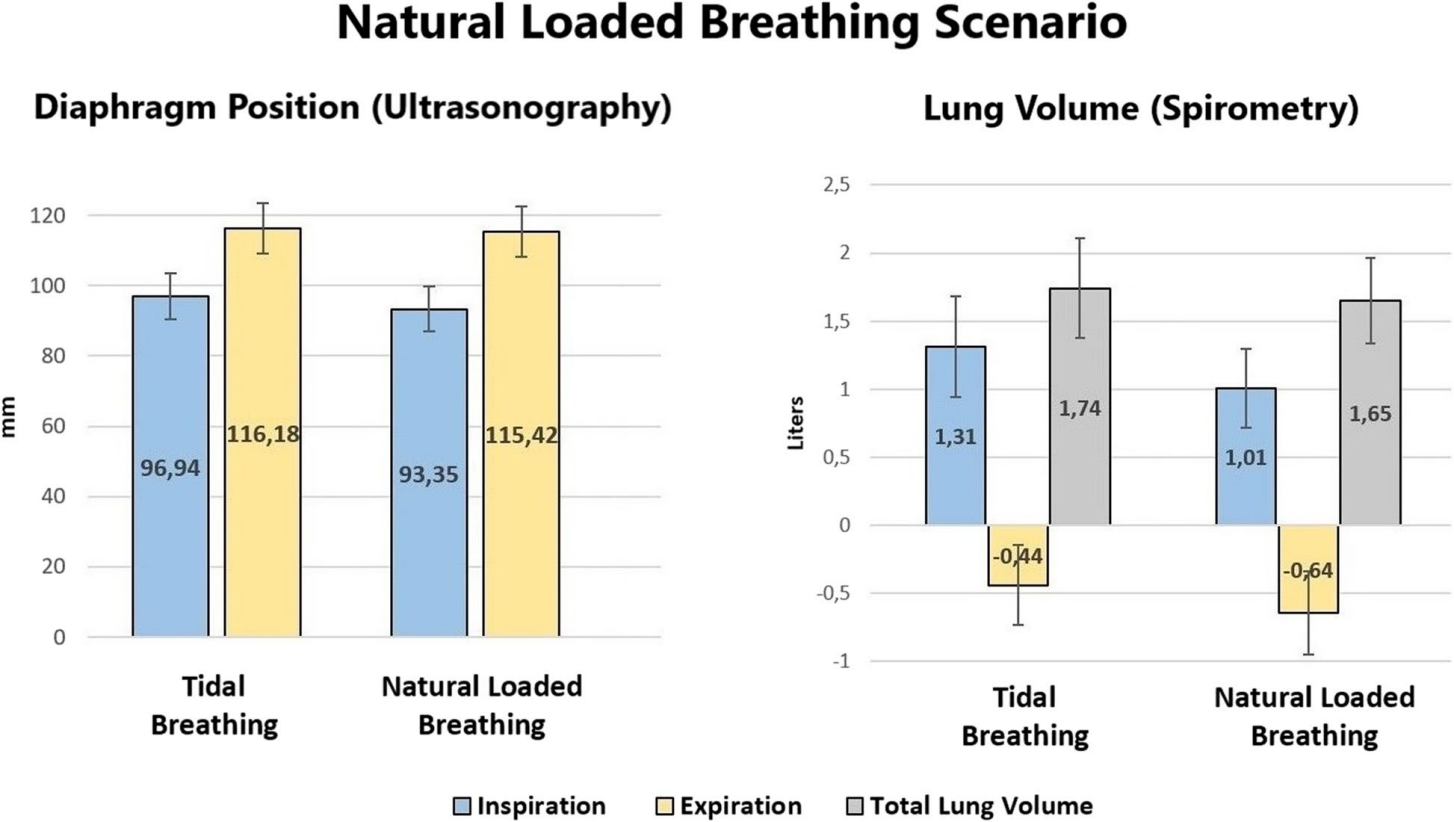
Diaphragm position (mm) and lung volume (L) during natural loaded breathing scenario (Mean ± Standard Deviation)

**Fig. 4:**
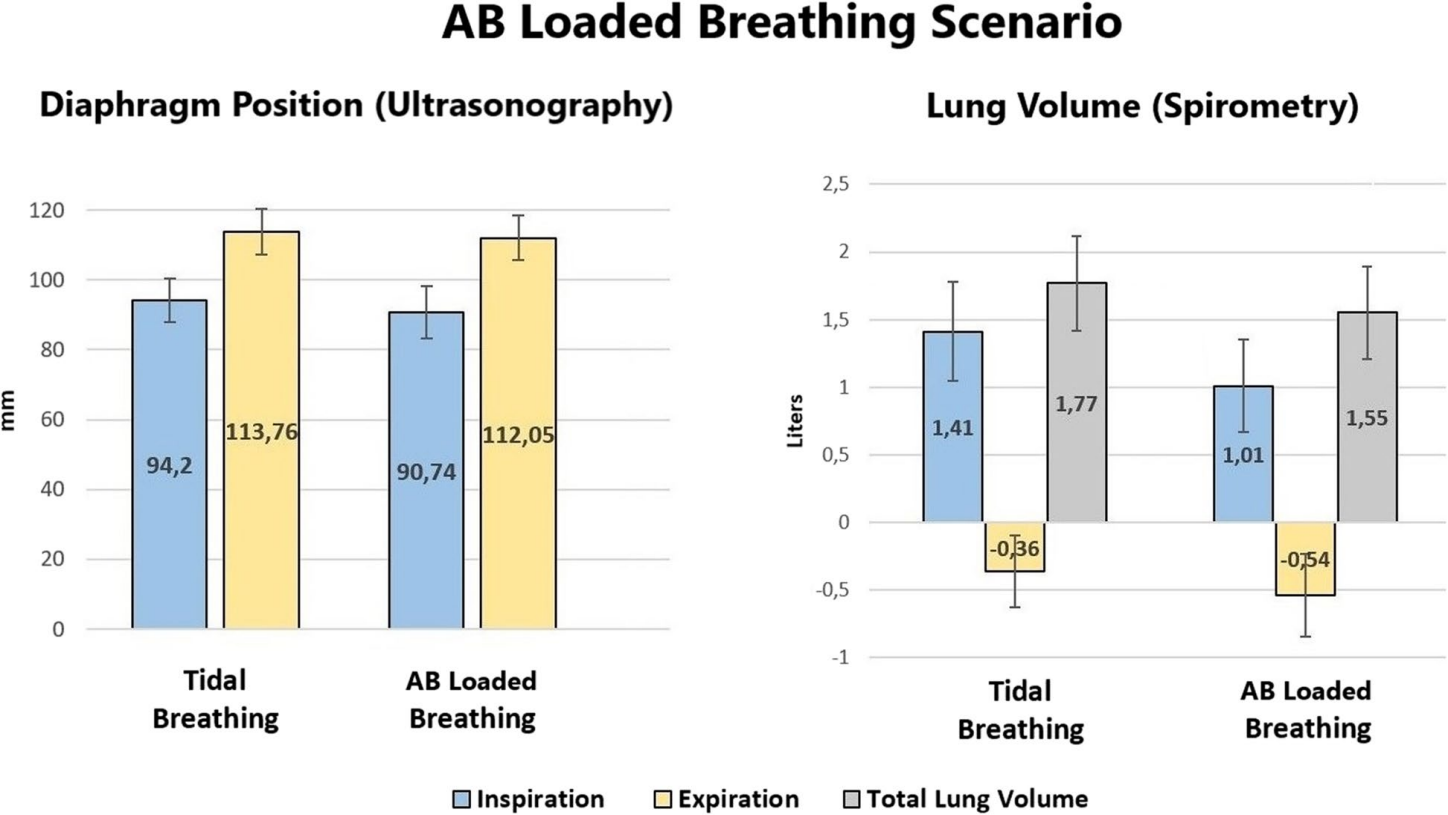
Diaphragm position (mm) and lung volume (L) during AB loaded breathing scenario (Mean ± Standard Deviation)

Table 4 displays the means ± standard deviation (SD), mean differences, and outcomes of each scenario. These data are presented graphically in Figs. 3 and 4. During the natural loaded breathing scenario, ultrasonography demonstrated the inspiratory position of the diaphragm (mm) when holding the weight was significantly lower, i.e., more caudal, compared to tidal inspiration (*t*(30) = 4.83, *p* < 0.001), but not for the expiratory position (*t*(30) = 1.31, *p* = 0.101). Spirometry values demonstrated that the lung volume (L) at the end of inspiration when holding the weight was significantly lower than the end of tidal inspiration (*t*(30) = 4.53, *p* < 0.001), as well as the end of expiration when holding the weight (*t*(30) = 4.75, *p* < 0.001). Total lung volume, calculated as the difference between the average inspiratory and expiratory values, was lower when holding the weight but not enough to be statistically significant (*t*(30) = 1.65, *p* = 0.06). During the AB loaded breathing scenario, ultrasonography demonstrated the inspiratory position of the diaphragm (mm) when holding the weight with AB was significantly lower compared to tidal inspiration (*t*(30) = 3.08, *p* = 0.002), but not for the expiratory position (*t*(30) = 1.69, *p* = 0.05). Spirometry values demonstrated that the lung volume (L) at the end of inspiration when holding the weight with AB was significantly lower than the end of tidal inspiration (*t*(30) = 3.52., *p* < 0.001), as well as the end of expiration holding the weight with AB (*t*(30) = 3.01, *p* = 0.002). Total lung volume was also significantly lower when holding the weight with AB (*t*(30) = 2.18, *p* = 0.019).

**Table 4:** Changes in Ultrasonography values (mm), Spirometric values (L), and Lung volume (L) during different scenarios of holding a load equivalent to 20% body weight (Mean [Standard Deviation])

**N-LBS**^**a**^	**Measure**	**Tidal Breathing**	**Natural Loaded Breathing**	**Mean Difference**	**95% CI**	**Effect Size**	***P* Value**
Ultrasonography	Inspiration	96.94 (13.03)	93.35 (12.92)	3.58	(2.07, 5.09)	0.87	< .001*
	Expiration	116.18 (14.35)	115.42 (14.51)	0.76	(-.428, 1.95)	0.235	0.101
Spirometry	Inspiration	1.31 (0.74)	1.01 (0.58)	0.30	(0.16, 0.44)	0.81	< .001*
	Expiration	-0.44 (0.59)	-0.64 (0.61)	0.21	(0.12, 0.30)	0.85	< .001*
	Lung Volume	1.74 (0.73)	1.65 (0.63)	0.09	(-0.02, 0.21)	0.3	0.06
**AB-LBS**^**b**^	**Measure**	**Tidal Breathing**	**AB Loaded Breathing**	**Mean Difference**	**95% CI**	**Effect Size**	***P* V*alue***
Ultrasonography	Inspiration	94.20 (12.27)	90.74 (14.89)	3.46	(1.16, 5.75)	0.55	.002*
	Expiration	113.76 (12.92)	112.05 (12.93)	1.71	(-0.35, 3.77)	0.3	0.05
Spirometry	Inspiration	1.41 (0.73)	1.01 (0.69)	0.40	(0.17, 0.63)	0.63	< .001*
	Expiration	-0.36 (0.53)	-0.54 (0.61)	0.18	(0.06, 0.30)	0.56	.002*
	Lunge Volume	1.77 (0.70)	1.55 (0.68)	0.22	(0.01, 0.43)	0.39	.019*

Note: *mm* millimeter, *L* = Liter

^a^Subject performs two tidal breaths, then holds the weight and performs two additional breaths with spontaneous abdominal muscle activity

^b^Subject voluntarily contracts abdominal muscles prior to holding weight, followed by two breaths

Note: *N-LBS=*Natural loaded breathing scenario

*AB-LBS *Abdominal bracing —loaded breathing scenario

Effect size = calculated Cohen's d

^*^Statistically significant difference observed (Bonferroni Correction *P* < 0.025)

## Discussion

This study has demonstrated that total lung volumes may be reduced during lifting a load compared to tidal breathing, although diaphragm movement increases. We assume that this is due to an increase in the stiffness of the chest by the insertional action of the abdominal muscles on its lower part [46], since a significant decrease in total lung volume occurred only when the abdominal muscles were voluntarily contracted. Contrary to our hypothesis, these findings imply that there is no greater contribution of the EIMs to ventilation during lifting tasks, but the expiratory muscles are more involved instead. The data presented in Table 4 show that when lifting weight, inspiratory volume is being reduced, even though the diaphragm reaches a more inspiratory (caudal) position compared to tidal breathing. Conversely, the expiratory volume increases while lifting weight, even though the diaphragm reaches the same expiratory position as during tidal breathing. This further supports the theory of greater involvement of the expiratory muscles. Such results may seem opposing to the findings of Hagins & Lamberg [47–49], who reported that whole-body lifting tasks resulted in a significant increase in inspiratory volume. However, these studies did not compare tidal and loaded breathing but only investigated breathing behavior during lifting a load. Since inspiration was identified in studies by Hagins & Lamberg before lift-off, inspiratory volume was thus increased.

Considering that the diaphragm plays a crucial role both in breathing and spinal stabilization [6, 8], its insufficient respiratory function can affect the postural function and vice versa. This is supported by several studies that showed the reduced respiratory or postural-respiratory movement of the diaphragm in patients with low back pain (LBP) [41, 50, 51], which may be associated with the decreased magnitude of the force the diaphragm is capable to exert [52]. Some previous studies suggest that lifting weights could improve diaphragm strength. DePalo et al. [53] demonstrated that progressive, graduated training of biceps curls and sit-ups for 16 weeks led to diaphragmatic hypertrophy and an increase in maximal inspiratory pressure. In addition, subjects who trained with weights had greater diaphragm thickness and achieved greater maximal inspiratory pressures compared to those who were untrained [54]. On the other hand, the controls in these two studies [53, 54] were non-training, so it is unclear whether these effects were caused by a specific type of workout or exercise in general. Janssens et al. [55, 56] also found that individuals with LBP were more prone to diaphragmatic fatigue compared to healthy controls. Even training the inspiratory muscles in LBP patients reduced pain intensity. This may help explain the association found between the presence of some respiratory disorders and LBP [57, 58].

Patients with chronic respiratory diseases often have lower diaphragmatic excursions than healthy controls [59–62]. Many of these patients suffer from dyspnea and associated exercise intolerance [63–66]. From the patient's perspective, dyspnea is a major factor that impairs the quality of life in chronic respiratory disorders, such as chronic obstructive pulmonary disease (COPD) [67]. An increase in dyspnea in COPD patients was found to be related to reduced exercise capacity and smaller diaphragmatic excursions [68]. Although resistance training is generally beneficial and recommended for COPD patients [69, 70], postural training of the diaphragm using lifting weights may not be advisable. For upper-body resistance training, including weight-lifting, studies have found no significant benefit in improving dyspnea and changes in maximal inspiratory pressure in patients with COPD [71, 72]. Moreover, the use of the upper limbs during different tasks can overload the EIMs and lead to dyspnea in these patients [73]. Our results demonstrated slight decreases in lung volume when lifting a load, but not statistically significant. However, when using AB during resistance training, consideration should be given to the fact that reduced lung volumes may contribute to the development of dyspnea in patients with respiratory disease.

The current study also shows a strong positive correlation between diaphragmatic movement and lung volumes during individual postural-respiratory maneuvers, such as tidal breathing, natural loaded breathing, and AB loaded breathing. This suggests that even during loaded breathing, the diaphragm is responsible for the majority of the inspired volume. However, this correlation does not fully explain why the overall lung volume decreased when lifting a load. As discussed above, we presume that lung volumes may be reduced by the postural engagement of other trunk muscles. We also observed a significant reduction in lung volume occurring during lifting a load with the simultaneous AB. In a related article [35], we reported that adding the AB maneuver during lifting weight results in a twofold increase in abdominal wall tension (AWT) compared to the spontaneous contraction of the abdominal muscles. Since the magnitude of AWT and IAP are strongly correlated [74], resistance to caudal diaphragmatic movement increases during AB. This suggests that adding AB when lifting a load can improve not only spinal stability but also the strength of the expiratory muscles as well as the diaphragm. Further research is needed to determine the effect of postural-respiratory training with or without AB on diaphragm thickness, movement, and strength in both healthy individuals and patients with LBP.

Our study has several limitations. Firstly, all subjects were healthy young volunteers, so the findings cannot be interpreted for a population of elderly individuals or those with respiratory disorders or musculoskeletal dysfunction. Secondly, it is unknown whether the same results would be confirmed for lifting heavier loads than 20% of body weight. In the present study, the weight could not be increased much more, as then the lifting would cause adverse body movement that would affect the ultrasonographic imaging of the diaphragm. Further research is needed to determine how heavier loads influence diaphragm movement as well as lung volumes. Lastly, we have not monitored the activity of the extra-diaphragmatic respiratory muscles while performing postural-respiratory maneuvers; thus, we can only speculate on their involvement during loaded breathing.

## Conclusion

Postural-respiratory contraction of the trunk muscles may reduce lung volume despite an increase in diaphragmatic motion during lifting a load. For patients at risk of dyspnea, it should be taken into account that lung volumes decreased significantly during loaded breathing with abdominal bracing. Still, strong correlations were found between lung volume and diaphragm movement for all types of breathing; suggesting major contributions of the diaphragm to respiration even during postural loading tasks.

## Data Availability

The datasets used and analyzed during this study are available in the Figshare repository. File 1. De-identified dataset for N-LBS. https://figshare.com/articles/dataset/File_1_De-identified_dataset_for_N-LBS_xlsx/22774892. File 2. De-identified dataset for AB-LBS. https://figshare.com/articles/dataset/File_2_De-identified_dataset_for_AB-LBS_xlsx/22774889.

## Abbreviations

AB: Abdominal bracing
AB-LBS: Abdominal bracing—loaded breathing scenario
AWT: Abdominal wall tension
BTPS: Body temperature and ambient pressure saturated with water vapour
COPD: Chronic obstructive pulmonary disease
EIMs: Extra-diaphragmatic inspiratory muscles
IAP: Intra-abdominal pressure
LBP: Low back pain
N-LBS: Natural loaded breathing scenario
WHtR: Waist to height ratio

## References

[1] Saboisky J, Gorman R, De Troyer A, Gandevia S, Butler J (2007). Differential activation among five human inspiratory motoneuron pools during tidal breathing. J Appl Physiol.

[2] Ratnovsky A, Elad D (2005). Anatomical model of the human trunk for analysis of respiratory muscles mechanics. Respir Physiol Neurobiol.

[3] Sant'Ambrogio G, Decandia M, Provini L (1966). Diaphragmatic contribution to respiration in the rabbit. J Appl Physiol.

[4] Mognoni P, Saibene F, Sant'Ambrogio G (1969). Contribution of the diaphragm and the other inspiratory muscles to different levels of tidal volume and static inspiratory effort in the rabbit. J Physiol.

[5] Sant'ambrogio G, Camporesi E (1973). Contribution of various inspiratory muscles to ventilation and the immediate and distant effect of diaphragmatic paralysis. Acta Neurobiol Exp (Wars).

[6] Hodges P, Gandevia S (2000). Changes in intra-abdominal pressure during postural and respiratory activation of the human diaphragm. J Appl Physiol.

[7] Hodges P, Gandevia S (2000). Activation of the human diaphragm during a repetitive postural task. J Physiol.

[8] Hodges P, Martin Eriksson A, Shirley D, C Gandevia S. Intra-abdominal pressure increases stiffness of the lumbar spine. Journal of Biomechanics. 2005; 38: 1873–1880. doi:10.1016/j.jbiomech.2004.08.016. 10.1016/j.jbiomech.2004.08.016 16023475

[9] Kolar P, Sulc J, Kyncl M, Sanda J, Neuwirth J, Bokarius A (2010). Stabilizing function of the diaphragm: dynamic MRI and synchronized spirometric assessment. J Appl Physiol.

[10] Hodges P, Kaigle Holm A, Holm S, Ekström L, Cresswell A, Hansson T (2003). Intervertebral Stiffness of the Spine Is Increased by Evoked Contraction of Transversus Abdominis and the Diaphragm. In Vivo Porcine Studies Spine.

[11] Danon J, Druz W, Goldberg N, Sharp J (1979). Function of the isolated paced diaphragm and the cervical accessory muscles in C1 quadriplegics. Am Rev Respir Dis.

[12] Strohl K, Mead J, Banzett R, Lehr J, Loring S, O’Cain C (1984). Effect of Posture on Upper and Lower Rib Cage Motion and Tidal Volume during Diaphragm Pacing. Am Rev Respir Dis.

[13] Cresswell A, Thorstensson A (1994). Changes in intra-abdominal pressure, trunk muscle activation and force during isokinetic lifting and lowering. Eur J Appl Physiol.

[14] Vera-Garcia F, Brown S, Gray J, McGill S (2006). Effects of different levels of torso coactivation on trunk muscular and kinematic responses to posteriorly applied sudden loads. Clin Biomech.

[15] Ishida H, Suehiro T, Kurozumi C, Watanabe S (2016). Comparison between the effectiveness of expiration and abdominal bracing maneuvers in maintaining spinal stability following sudden trunk loading. J Electromyogr Kinesiol.

[16] Stanton T, Kawchuk G (2008). The Effect of Abdominal Stabilization Contractions on Posteroanterior Spinal Stiffness. Spine.

[17] Larivière C, Boucher J, Mecheri H, Ludvig D (2019). Maintaining Lumbar Spine Stability: A Study of the Specific and Combined Effects of Abdominal Activation and Lumbosacral Orthosis on Lumbar Intrinsic Stiffness. J Orthop Sports Phys Ther.

[18] Coenen P, Campbell A, Kemp-Smith K, O'Sullivan P, Straker L (2017). Abdominal bracing during lifting alters trunk muscle activity and body kinematics. Appl Ergon.

[19] Panjabi M (2003). Clinical spinal instability and low back pain. J Electromyogr Kinesiol.

[20] Willardson J. Core Stability Training: Applications to Sports Conditioning Programs. The Journal of Strength and Conditioning Research. 2007; 21: R-20255. doi:10.1519/R-20255.1. 10.1519/R-20255.1 17685697

[21] Hibbs A, Thompson K, French D, Wrigley A, Spears I (2008). Optimizing Performance by Improving Core Stability and Core Strength. Sports Med.

[22] Grenier S, McGill S (2007). Quantification of Lumbar Stability by Using 2 Different Abdominal Activation Strategies. Arch Phys Med Rehabil.

[23] Bolton C, Bevan-Smith E, Blakey J, Crowe P, Elkin S, Garrod R, et al. British Thoracic Society guideline on pulmonary rehabilitation in adults: accredited by NICE. Thorax. 2013; 68: ii1-ii30. doi:10.1136/thoraxjnl-2013-203808. 10.1136/thoraxjnl-2013-203808 23880483

[24] Garvey C, Bayles M, Hamm L, Hill K, Holland A, Limberg T (2016). Pulmonary Rehabilitation Exercise Prescription in Chronic Obstructive Pulmonary Disease. J Cardiopulm Rehabil Prev.

[25] Jimeno-Almazán A, Franco-López F, Buendía-Romero Á, Martínez-Cava A, Sánchez-Agar J, Sánchez-Alcaraz Martínez B (2022). Rehabilitation for post-COVID -19 condition through a supervised exercise intervention: A randomized controlled trial. Scand J Med Sci Sports.

[26] Sandberg J, Ekström M, Börjesson M, Bergström G, Rosengren A, Angerås O, et al. Underlying contributing conditions to breathlessness among middle-aged individuals in the general population: a cross-sectional study. BMJ Open Respiratory Research. 2020; 7. doi:10.1136/bmjresp-2020-000643. 10.1136/bmjresp-2020-000643 PMC7520902 32978243

[27] Nalbandian A, Sehgal K, Gupta A, Madhavan M, McGroder C, Stevens J (2021). Post-acute COVID-19 syndrome. Nat Med.

[28] Pierce J, Shen Q, Cintron S, Hiebert J (2022). Post-COVID-19 Syndrome. Nurs Res.

[29] Gilman S, Banzett R (2009). Physiologic changes and clinical correlates of advanced dyspnea. Curr Opin Support Palliat Care.

[30] Houston J, Angus R, Cowan M, McMillan N, Thomson N (1994). Ultrasound assessment of normal hemidiaphragmatic movement: relation to inspiratory volume. Thorax.

[31] Hodges P, Heijnen I, Gandevia S (2001). Postural activity of the diaphragm is reduced in humans when respiratory demand increases. J Physiol.

[32] Kantarci F, Mihmanli I, Demirel M, Harmanci K, Akman C, Aydogan F (2004). Normal Diaphragmatic Motion and the Effects of Body Composition. J Ultrasound Med.

[33] Noh D, Lee J, You J (2014). Diaphragm Breathing Movement Measurement using Ultrasound and Radiographic Imaging: A Concurrent Validity. Bio-Med Mater Eng.

[34] Scarlata S, Mancini D, Laudisio A, Benigni A, Antonelli IR (2018). Reproducibility and Clinical Correlates of Supine Diaphragmatic Motion Measured by M-Mode Ultrasonography in Healthy Volunteers. Respiration.

[35] Sembera M, Busch A, Kobesova A, Hanychova B, Sulc J, Kolar P. Postural-respiratory function of the diaphragm assessed by M-mode ultrasonography. PLOS ONE. 2022; 17. doi:10.1371/journal.pone.0275389. 10.1371/journal.pone.0275389 PMC9550028 36215306

[36] Houston J, Morris A, Howie C, Reid J, McMillan N (1992). Technical report: Quantitative assessment of diaphragmatic movement — A reproducible method using ultrasound. Clin Radiol.

[37] Ayoub J, Cohendy R, Dauzat M, Targhetta R, De La Coussaye J, Bourgeois J (1997). Non-invasive quantification of diaphragm kinetics using m-mode sonography. Can J Anaesth.

[38] Boussuges A, Gole Y, Blanc P (2009). Diaphragmatic Motion Studied by M-Mode Ultrasonography. Chest.

[39] Sarwal A, Walker F, Cartwright M (2013). Neuromuscular ultrasound for evaluation of the diaphragm. Muscle Nerve.

[40] Kolar P, Neuwirth J, Sanda J, Suchanek V, Svata Z, Pivec M (2009). Analysis of diaphragm movement during tidal breathing and during its activation while breath holding using MRI synchronized with spirometry. Physiol Res.

[41] Kolar P, Sulc J, Kyncl M, Sanda J, Cakrt O, Andel R (2012). Postural Function of the Diaphragm in Persons With and Without Chronic Low Back Pain. J Orthop Sports Phys Ther.

[42] Kim H. Statistical notes for clinical researchers: assessing normal distribution (2) using skewness and kurtosis. Restorative Dentistry & Endodontics. 2013; 38. doi:10.5395/rde.2013.38.1.52. 10.5395/rde.2013.38.1.52 PMC3591587 23495371

[43] Field A (2017). Discovering Statistics Using IBM SPSS Statistics.

[44] Koo T, Li M (2016). A Guideline of Selecting and Reporting Intraclass Correlation Coefficients for Reliability Research. J Chiropr Med.

[45] Cohen J (1988). Statistical power analysis for the behavioral sciences.

[46] D'Angelo E, Prandi E, Robatto F, Petitjean M, Bellemare F (1996). Insertional action of the abdominal muscles in rabbits and dogs. Respir Physiol.

[47] Hagins M, Lamberg E (2006). Natural breath control during lifting tasks: effect of load. Eur J Appl Physiol.

[48] Lamberg E, Hagins M (2010). Breath control during manual free-style lifting of a maximally tolerated load. Ergonomics.

[49] Hagins M, Lamberg E (2011). Individuals with Low Back Pain Breathe Differently Than Healthy Individuals During a Lifting Task. J Orthop Sports Phys Ther.

[50] Vostatek P, Novak D, Rychnovsky T, Rychnovska S, Yue J. Diaphragm Postural Function Analysis Using Magnetic Resonance Imaging. PLoS ONE. 2013; 8. doi:10.1371/journal.pone.0056724. 10.1371/journal.pone.0056724 PMC3597716 23516397

[51] Mohan V, Paungmali A, Sitilerpisan P, Hashim U, Mazlan M, Nasuha T (2018). Respiratory characteristics of individuals with non-specific low back pain: A cross-sectional study. Nurs Health Sci.

[52] Koco E, Soilemezi E, Sotiriou P, Savvidou S, Tsagourias M, Pnevmatikos I, et al. Ultrasonographic assessment of diaphragmatic contraction and relaxation properties: correlations of diaphragmatic displacement with oesophageal and transdiaphragmatic pressure. BMJ Open Respiratory Research. 2021; 8. doi:10.1136/bmjresp-2021-001006. 10.1136/bmjresp-2021-001006 PMC8461713 34556491

[53] DePalo V, Parker A, Al-Bilbeisi F, McCool F (2004). Respiratory muscle strength training with nonrespiratory maneuvers. J Appl Physiol.

[54] McCool F, Conomos P, Benditt J, Cohn D, Sherman C, Hoppin F (1997). Maximal inspiratory pressures and dimensions of the diaphragm. Am J Respir Crit Care Med.

[55] Janssens L, Brumagne S, McConnell A, Hermans G, Troosters T, Gayan-Ramirez G (2013). Greater diaphragm fatigability in individuals with recurrent low back pain. Respir Physiol Neurobiol.

[56] Janssens L, McConnell A, Pijnenburg M, Claeys K, Goossens N, Lysens R (2015). Inspiratory Muscle Training Affects Proprioceptive Use and Low Back Pain. Med Sci Sports Exerc.

[57] Beeckmans N, Vermeersch A, Lysens R, Van Wambeke P, Goossens N, Thys T (2016). The presence of respiratory disorders in individuals with low back pain: A systematic review. Man Ther.

[58] Rasmussen-Barr E, Magnusson C, Nordin M, Skillgate E (2019). Are respiratory disorders risk factors for troublesome low-back pain? A study of a general population cohort in Sweden. Eur Spine J.

[59] Dos Santos YW, Paulin E, Shibao S, Chammas M, Salge J, Ribeiro M (2008). Air trapping: The major factor limiting diaphragm mobility in chronic obstructive pulmonary disease patients. Respirology.

[60] Boccatonda A, Decorato V, Cocco G, Marinari S, Schiavone C. Ultrasound evaluation of diaphragmatic mobility in patients with idiopathic lung fibrosis: a pilot study. Multidisciplinary Respiratory Medicine. 2019; 14. doi:10.1186/s40248-018-0159-y. 10.1186/s40248-018-0159-y PMC6330497 30651988

[61] Tanriverdi A, Savci S, Mese M, Gezer N, Kahraman B, Sevinc C (2021). Diaphragmatic Ultrasound in Non-Cystic Fibrosis Bronchiectasis: Relationship to Clinical Parameters. Ultrasound Med Biol.

[62] Ishak S, Sakr H (2022). Diaphragmatic thickness and excursion by lung ultrasound in pediatric chronic pulmonary diseases. J Ultrasound.

[63] Cropp G, Pullano T, Cerny F, Nathanson I (1982). Exercise tolerance and cardiorespiratory adjustments at peak work capacity in cystic fibrosis. Am Rev Respir Dis.

[64] Laveneziana P, Parker C, O’Donnell D (2007). Ventilatory constraints and dyspnea during exercise in chronic obstructive pulmonary disease. Appl Physiol Nutr Metab.

[65] Vainshelboim B, Fox B, Oliveira J, Kramer M (2015). Exercise training in idiopathic pulmonary fibrosis. Expert Rev Respir Med.

[66] José A, Ramos T, de Castro R, de Oliveira C, de Camargo A, Athanazio R (2018). Reduced Physical Activity With Bronchiectasis. Respir Care.

[67] Ries A (2006). Impact of Chronic Obstructive Pulmonary Disease on Quality of Life: The Role of Dyspnea. Am J Med.

[68] Shiraishi M, Higashimoto Y, Sugiya R, Mizusawa H, Takeda Y, Fujita S, et al. Diaphragmatic excursion correlates with exercise capacity and dynamic hyperinflation in COPD patients. ERJ Open Research. 2020; 6. doi:10.1183/23120541.00589-2020. 10.1183/23120541.00589-2020 PMC7792831 33447614

[69] Strasser B, Siebert U, Schobersberger W (2013). Effects of resistance training on respiratory function in patients with chronic obstructive pulmonary disease: a systematic review and meta-analysis. Sleep and Breathing.

[70] Liao W, Chen J, Chen X, Lin L, Yan H, Zhou Y (2015). Impact of Resistance Training in Subjects With COPD: A Systematic Review and Meta-Analysis. Respir Care.

[71] Janaudis-Ferreira T, Hill K, Goldstein R, Robles-Ribeiro P, Beauchamp M, Dolmage T (2011). Resistance Arm Training in Patients With COPD. Chest.

[72] McAuley M, McAuley E, Kapella M, Collins E, Alex C, Berbaum M, Larson J. Upper-Body Resistance Training and Self-Efficacy Enhancement in COPD. Journal of Pulmonary & Respiratory Medicine. 2012; 02. doi:10.4172/2161-105X.S9-001. 10.4172/2161-105X.S9-001 PMC3975911 24707449

[73] Celli B, Rassulo J, Make B (1986). Dyssynchronous Breathing during Arm but Not Leg Exercise in Patients with Chronic Airflow Obstruction. N Engl J Med.

[74] Novak J, Jacisko J, Busch A, Cerny P, Stribrny M, Kovari M, et al. Intra-abdominal pressure correlates with abdominal wall tension during clinical evaluation tests. Clinical Biomechanics. 2021; 88. doi:10.1016/j.clinbiomech.2021.105426. 10.1016/j.clinbiomech.2021.105426 34303067

